# Cholesterol activates the Wnt/PCP-YAP signaling in SOAT1-targeted treatment of colon cancer

**DOI:** 10.1038/s41420-021-00421-3

**Published:** 2021-02-26

**Authors:** Huanji Xu, Hongwei Xia, Sheng Zhou, Qiulin Tang, Feng Bi

**Affiliations:** Department of Medical Oncology, Cancer Center and Laboratory of Molecular Targeted Therapy in Oncology, West China Hospital, Sichuan University, 610041 Chengdu, Sichuan Province China

**Keywords:** Lipid signalling, Cancer metabolism, Targeted therapies

## Abstract

Intracellular free cholesterol can be converted to cholesteryl ester and stored as lipid droplets through SOAT1-mediated esterification. Compelling evidence implicate targeting SOAT1 as a promising therapeutic strategy for cancer management. Herein, we demonstrate how targeting SOAT1 promotes YAP expression by elevating cellular cholesterol content in colon cancer cells. Results revealed that cholesterol alleviates the inhibitory effect of LRP6 on the Wnt/PCP pathway by impeding the interaction of LRP6 with FZD7. Subsequently, FZD7-mediated PCP signaling directly elevated YAP expression by activating RhoA. Nystatin-mediated cholesterol sequestration significantly inhibited YAP expression under SOAT1 inhibition. Moreover, nystatin synergized with the SOAT1 inhibitor avasimibe in suppressing the viability of colon cancer cells in vitro and in vivo. The present study provides new mechanistic insights into the functions of cholesterol metabolism on growth signaling pathways and implicates a novel strategy for cholesterol metabolic-targeted treatment of colon cancers.

## Introduction

Cancer cells reprogram metabolic pathways to meet their abnormal demands for proliferation and survival^1^. Cholesterol, regarded as the basic component of the plasma membrane, regulates its integrity and physical properties^2^. Cancer cells acquire cholesterol via endogenous biosynthesis and exogenous uptake. Several cancers drive reprogrammed cholesterol biosynthesis and uptake^3^. Enhancing cholesterol availability, either in form of a diet supplement or stimulating its biosynthesis, promotes tumorigenesis and tumor progression of colon cancer, breast cancer, and liver cancer^4–6^. However, information on the direct effect of cholesterol on oncogenic signaling pathways in cancer remains scanty.

Through sterol O-acyltransferase 1/2 (SOAT1/2)-mediated esterification, excess cellular cholesterol is converted into cholesteryl ester and stored as lipid droplets^7^. SOAT1 is ubiquitously expressed in most tissues, whereas SOAT2 expression is restricted to the liver and small intestine^8^. In recent years, a wealth of studies has revealed that SOAT1 is highly expressed in numerous tumors, among them, pancreatic carcinoma, hepatocellular carcinoma (HCC), and prostatic cancer^3,9,10^. The transcription factor, SREBPs, precisely regulate intracellular cholesterol levels. Notably, a slight rise in cholesterol content would induce feedback inhibition of SREBPs activities^11^. Further reports demonstrated that targeting SOAT1 could inhibit SREBP1-mediated fatty acid metabolism by increasing intracellular free cholesterol level, consequently inhibiting tumor growth^10,12^. Additionally, elevated membrane cholesterol levels induced by SOAT1 inhibition could enhance the killing effect of CD8+ T cells on melanoma^13^. Following these previous findings, SOAT1 is a promising therapeutic target for cancer management. Therefore, it is indispensable to explore the oncogenic signaling pathways affected by SOAT1 inhibition and approaches to sensitize cells to SOAT1-targeted therapy in cancer.

Hippo/YAP pathway plays a critical role in regulating organ size, tissue regeneration, tumor formation, and stem cell function^14^. Excessive YAP activation is implicated in various human malignant tumors and drug resistance^15^. Herein, we demonstrate that targeting SOAT1 promotes YAP expression by elevating cellular cholesterol content in colon cancer cells. Besides, cellular cholesterol functions via the PCP (planar cell polarity)/RhoA pathway to regulate YAP expression. Nystatin-mediated cholesterol sequestration could sensitize colon cancer cells to SOAT1 ablation. Notably, these findings could avail new mechanistic insights into the role of cholesterol in the Hippo/YAP pathway, uncover the basis of intrinsic resistance to SOAT1-targeted therapy, and suggest a novel approach for cholesterol metabolic-targeted treatment of colon cancers.

## Results

### SOAT1 is highly expressed in colon cancer

**Fig. 1:**
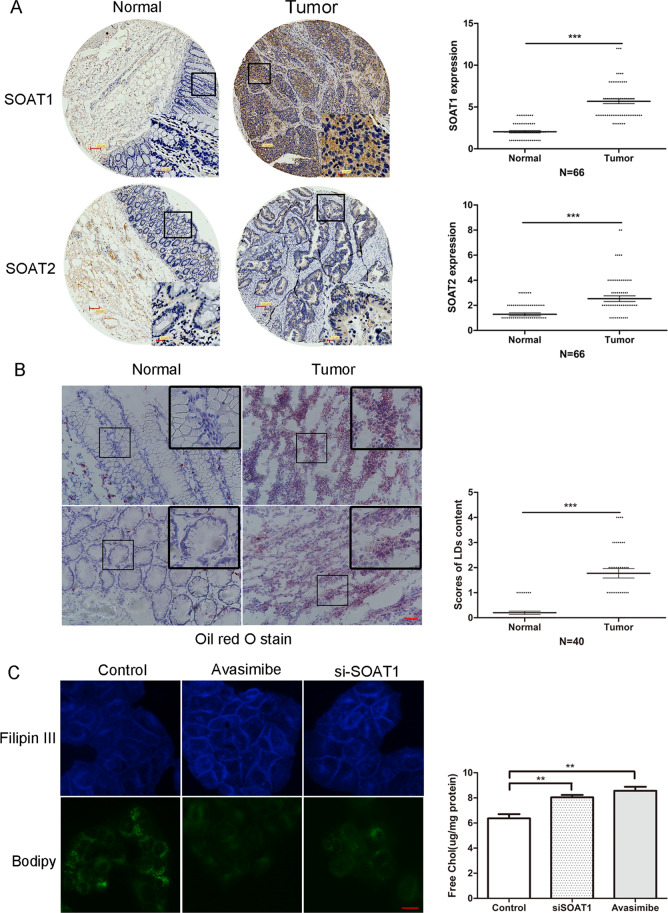
SOAT1 is highly expressed in colon cancer. **A** Representative pictures of SOAT1/2 staining in clinical samples of normal and colon cancer tissues. The graphs indicate the IHC scores of SOAT1/2 expression. ***p < 0.001 using Student’s *t* test (two-tailed). Scale bar: 100/15 μm. **B** Detecting lipid droplets in 40 pairs of colon cancer tissues and normal tissues by Oil Red O staining. The graph indicates statistical result of lipid droplet content scores. ***p < 0.001 using Student’s *t* test (two-tailed). Scale bar: 50 μm. **C** SW1116 cells were transfected with SOAT1 siRNA for 48 h or treated with avasimibe 10 μM for 16 h, and then stained by Filipin III. The graph indicates the quantitative result of intracellular cholesterol content. **p < 0.01 using Student’s *t* test (two-tailed). Scale bar: 20 μm; SW1116 were transfected with SOAT1 interfering RNA for 72 h or treated with avasimibe 10 μM for 24 h. Bodipy 493/503 was used for lipid droplets staining.

Through immunohistochemistry (IHC) analysis, SOAT1/2 levels were assessed in a colon cancer tissue microarray (TMA) comprising 66 matched pairs of carcinoma and normal tissue samples. Compared to normal tissues, SOAT1 expression was significantly higher in colon tumor tissues. Besides, in colon cancers, much lower SOAT2 was expressed than SOAT1 (Fig. 1A). As cholesteryl ester is stored in lipid droplets (LDs), we performed Oil Red O staining to detect LDs in 40 matched pairs of colon cancer and normal tissues. Notably, LDs were highly prevalent in colon cancer tissues, but nearly undetectable in normal tissues (Fig. 1B). Furthermore, SOAT1 was inhibited by siRNA or avasimibe in SW1116 cells to elucidate whether it regulated LDs in colon cancer. LDs staining by Bodipy 493/503 demonstrated significantly decreased LDs levels. Moreover, cholesterol staining using Filipin III and cholesterol detection kit revealed high intracellular cholesterol content (Fig. 1C). Similar findings were reported in previous studies on other tumor types^9,10,12^, whereby SOAT1-mediated cholesterol esterification was markedly enhanced in colon cancer.

### Targeting SOAT1 promotes YAP expression via cellular cholesterol in colon cancer cells

**Fig. 2:**
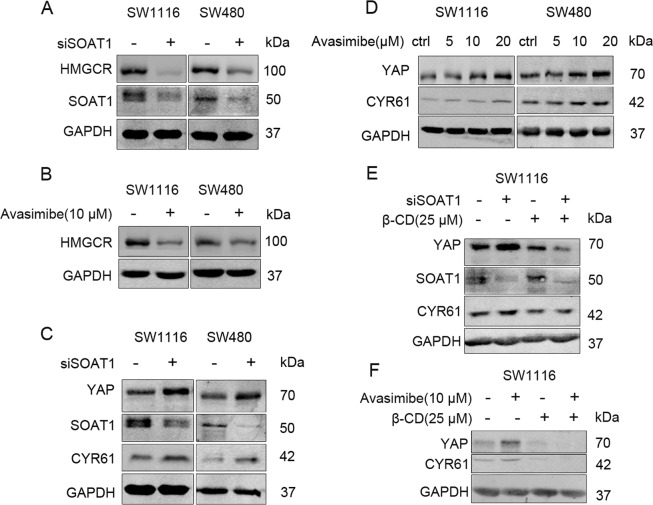
Targeting SOAT1 regulates YAP in a cholesterol-dependent manner in colon cancer cells. **A** SW1116 and SW480 cells were transfected with control siRNA or siSOAT1 for 72 h, respectively. **B** SW1116 and SW480 were incubated with DMSO or avasimibe (10 μM) for 24 h. **C** SW1116 and SW480 were transfected with negative control siRNA or SOAT1 siRNA for 48 h, respectively. **D** SW1116 and SW480 cells were treated with different concentration of avasimibe (5–20 μM) for 16 h. **E** SW1116 were transfected with negative control or SOAT1 siRNA for 48 h, and then treated with β-cyclodextrin (25 μM) for 4 h. **F** SW1116 cells were treated with DMSO or avasimibe (10 μM) for 12 h, and then β-cyclodextrin (25 μM) was incubated for 4 h. The expressions of HMGCR, YAP, and CYR61 were detected by Western Blot.

Elevated intracellular free cholesterol content could inhibit SREBPs activities^16^. Upon evaluating the effect of SOAT1 inhibition on cholesterol biosynthesis, we found that SOAT1 siRNA or avasimibe lowered the expression levels of HMGCR (Fig. 2A, B). As inhibition of the mevalonate (MVA) pathway could suppress YAP activity^17^, we assessed the YAP expression and activity post SOAT1 siRNA transfection or avasimibe treatment in SW480 and SW1116 cells. Of note, the expression levels of YAP and its downstream target CYR61 were rather elevated following SOAT1 inhibition (Fig. 2C, D).

Reports have implicated cholesterol as an unique signaling molecule, regulating multiple oncogenic pathways, including the canonical Wnt pathway and Hedgehog pathway^11,18–20^. To validate whether targeting SOAT1 potentially elevates intracellular free cholesterol content as a mechanism for regulating YAP expression, we used β-cyclodextrin (β-CD) to extract cholesterol from colon cancer cells. Results demonstrated that β-CD could reverse the elevated expression of YAP, induced by SOAT1 silencing or avasimibe (Fig. 2E, F). Thus, we deduced that targeting SOAT1 was dependent on cellular cholesterol to promote YAP expression in colon cancer cells.

### Cholesterol promotes YAP expression independent of LATS and β-Catenin in colon cancer cells

**Fig. 3:**
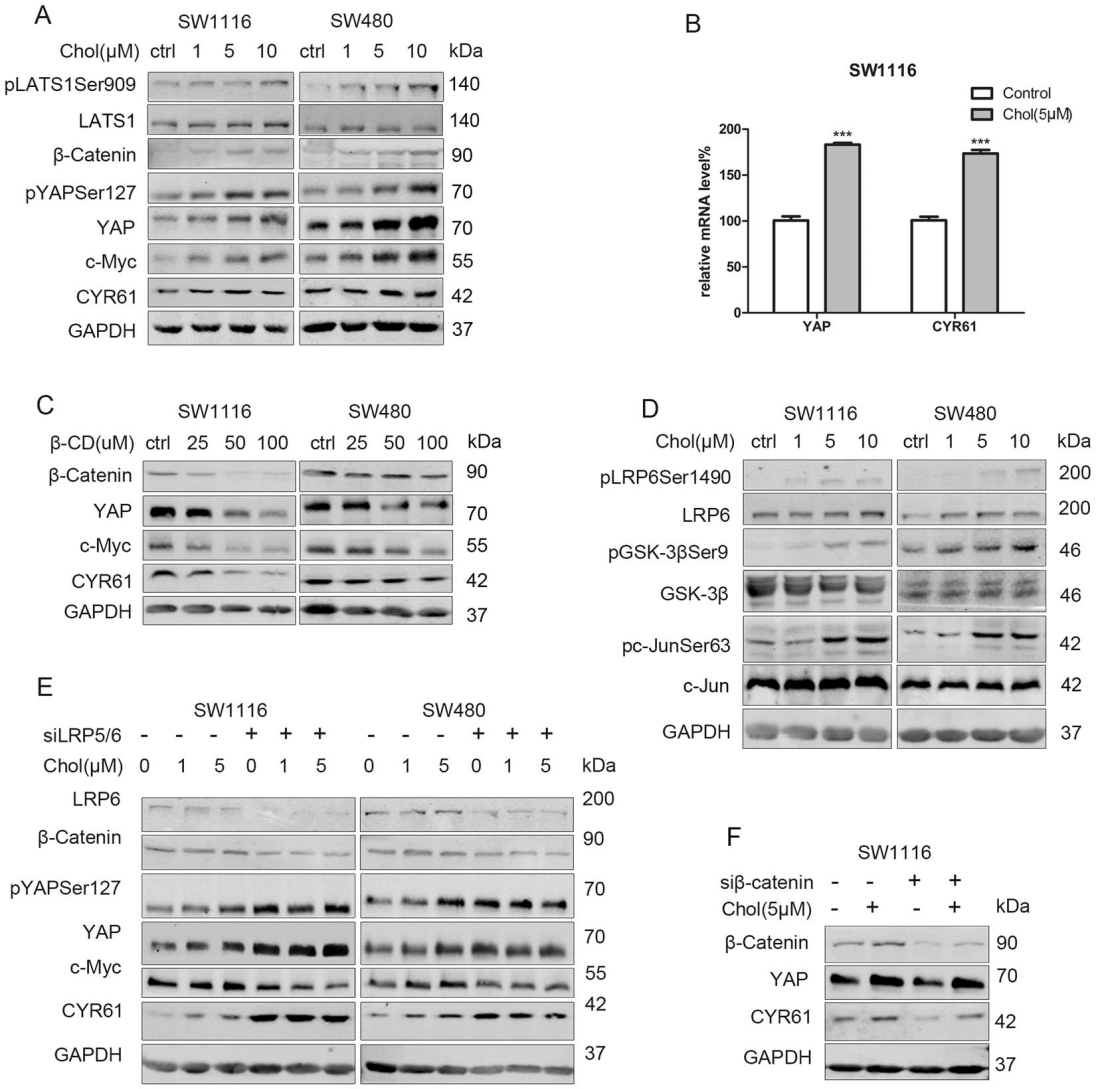
Cholesterol promotes YAP expression independent of LATS and β-Catenin in colon cancer cells. **A** SW1116 and SW480 were starved in serum-free medium for 24 h, and then treated with increasing concentration (1–10 μM) of cholesterol for 2 h. **B** SW1116 were starved for 24 h, and then treated with cholesterol (5 μM) for 2 h. The mRNA levels of YAP and CYR61 were detected by qPCR. ***p < 0.001 using Student’s *t* test (two-tailed). **C** Increasing concentration of β-cyclodextrin (25–100 μM) was incubated in SW1116 and SW480 cells for 4 h. (D): SW1116 and SW480 cells were starved for 24 h, incubated with increasing concentration of cholesterol (1–10 μM) for 2 h. **E** SW1116 and SW480 were transfected with negative control siRNA or LRP5/6 siRNA for 48 h, then starved for 24 h, and incubated with DMSO or cholesterol (1–5 μM) for 2 h. **F** SW1116 were transfected with negative siRNA or β-Catenin siRNA for 48 h, then starved for 24 h, and then incubated with DMSO or cholesterol (5 μM) for 2 h. Western blot analysis was performed to detect the expression of proteins.

To prove the potential regulatory role of cholesterol on YAP expression, we incubated SW1116 and SW480 cells with exogenous cholesterol. The result showed that the expression levels of YAP and CYR61 were increased (Fig. 3A, S1). Notably, the expressions of pLATS1 and pYAPSer127 were elevated upon stimulation with exogenous cholesterol, demonstrating that Hippo/LATS cascade was also activated (Fig. 3A). qPCR assays revealed that cholesterol elevated the mRNA levels of YAP and CYR61 (Fig. 3B). Based on these findings, cholesterol-regulated YAP expression at the transcriptional level rather than by inhibiting Hippo/LATS-mediated phosphorylation-dependent degradation. Also, the activation of the Hippo/LATS cascade is a potential feedback mechanism in response to YAP activation^21^. Introducing β-CD to directly eliminate cellular cholesterol could also lower YAP and CYR61 expressions. Thus, we suggested that cellular cholesterol could regulate YAP expression (Fig. 3C).

Previous studies had noted that cholesterol could directly activate the canonical Wnt pathway^18^; herein, we explored the effect of cholesterol on the canonical Wnt pathway under our experimental conditions. Notably, cholesterol elevated the expressions of β-Catenin and its downstream target c-Myc in SW1116 and SW480 cells (Fig. 3A, S1). Additionally, the activation markers of canonical Wnt signaling pLRP6Ser1490 and GSK-3βSer9 were up-regulated (Fig. 3D). LRP5/6 silencing reversed the high expression levels of β-Catenin and c-Myc (Fig. 3E), further demonstrating the potential role of cholesterol in canonical Wnt/β-Catenin pathway activation. β-Catenin/TCF4 complexes could bind the DNA enhancer element of the YAP gene, promoting YAP expression^22^. However, cholesterol still elevated YAP expression in the absence of β-Catenin in SW1116 cells (Fig. 3F, S2). Thus, we suggested that cholesterol promoted YAP expression via a Hippo/LATS and β-Catenin-independent mechanism in colon cancer cells.

### Cholesterol activates the FZD7-PCP pathway to promote YAP expression in colon cancer cells

**Fig. 4:**
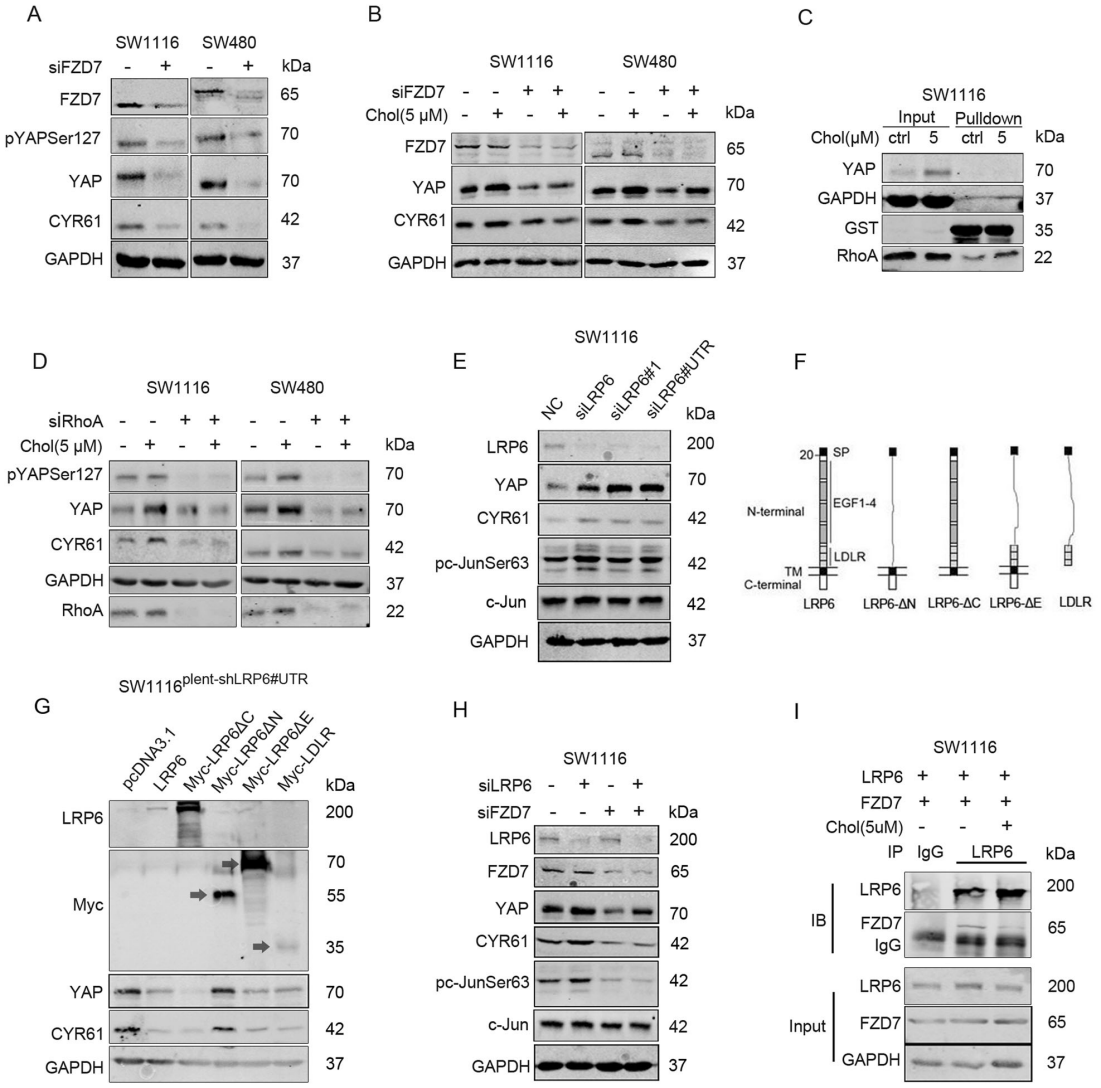
Cholesterol alleviates the inhibitory effect of LRP6 on FZD7-PCP pathway to promote YAP expression in colon cancer cells. **A** SW1116 and SW480 were transfected with negative control siRNA or FZD7 siRNA for 72 h, respectively. **B** SW1116 and SW480 were transfected with negative control siRNA or FZD7 siRNA for 48 h, and starved in serum-free medium for 24 h, then incubated with DMSO or cholesterol (5 μM) for 2 h, respectively. **C** SW1116 cells were starved for 24 h, then incubated with DMSO or cholesterol (5 μM) for 2 h. RhoA activity was detected by GST pull-down assay. **D** SW1116 and SW480 were transfected with negative control siRNA or RhoA siRNA for 48 h, then starved for 24 h, and incubated with DMSO or cholesterol (5 μM) for 2 h, respectively. **E** SW1116 were transfected with siLRP6, siLRP6#1, and siLRP6#UTR (targeting 5’-untranslated region of LRP6 mRNA) for 72 h. **F** Schematic diagram of LRP6 and LRP6 deletion mutants. **G** SW1116 stably expressing shLRP6#UTR was constructed by lentiviral vector. SW1116 stable cell line were transfected with control vector or LRP6 and deletion mutants vectors for 72 h. **H** SW1116 was transfected with negative control siRNA or siLRP6, in the presence or absence of FZD7 deletion. **I** SW1116 was transfected with vectors expressing LRP6 and FZD7 for 48 h, followed by starvation for 24 h, and then treated with cholesterol (5 μM) or DMSO for 2 h. Co-IP assay was performed to detect the interaction between LRP6 and FZD7.

An existing report found that the non-canonical Wnt/PCP pathway could be mediated by RhoA to promote the expression and activity of YAP^23^. To uncover the specific FZD receptor in the PCP pathway that potentially drives cholesterol-mediated regulation of YAP, the receptors FZD1/2/5/7/8 were silenced, respectively. Results revealed that FZD7 deletion, solely down-regulated YAP expression (Fig. 4A, S3), and reversed the effect of cholesterol on YAP expression (Fig. 4B, S4).

Cholesterol activated the activities of c-Jun and RhoA, two downstream proteins of the PCP pathway (Figs. 3D, 4C). Moreover, RhoA silencing reversed the up-regulation of YAP and CYR61, induced by cholesterol stimulation (Fig. 4D). Collectively, cholesterol potentially up-regulated YAP expression via the FZD7/PCP pathway activation.

### Cholesterol alleviates the inhibitory effect of LRP6 on the PCP pathway to promote YAP expression in colon cancer cells

Cholesterol activated canonical Wnt/β-Catenin signaling and non-canonical Wnt/PCP signaling simultaneously; thus, we supposed that it could induce Wnt protein secretion. However, cholesterol further up-regulated the expression of YAP and β-Catenin when pretreated with LGK974, an inhibitor for Wnt proteins secretion (Fig. S5). Thus, it was suggested that cholesterol regulation of YAP expression was independent of stimulated Wnt proteins secretion.

Of note, following LRP5/6 silencing, we found that YAP and CYR61 expressions were significantly up-regulated; also, the effect of cholesterol on YAP expression faded (Fig. 3E). We concluded that regulation of the PCP/YAP axis by cholesterol is may depend on LRP5/6.

To further establish the regulatory effect of LRP6 on YAP expression in colon cancer cells, three siRNAs with different sequences (siLRP6, siLRP6#1, and siLRP6#UTR) were administered to SW1116 cells, whereas siLRP6#UTR targeted the 5′-untranslated region (5′-UTR) of LRP6 mRNA. Notably, results demonstrated high expression levels of YAP and phosphorylated c-Jun following transfection with the three LRP6 siRNA, respectively (Fig. 4E).

To illuminate the underlying mechanism, a stable cell line SW1116^plent-shLRP6#UTR^ expressing siLRP6#UTR persistently was constructed. Vectors over-expressing LRP6 and LRP6 truncated mutants (LRP6-ΔC, LRP6-ΔN, LRP6-ΔE, and LDLR) were then transfected into SW1116^plent-shLRP6#UTR^ (Fig. 4F). Based on the results, recovering the expressions of LRP6, LRP6ΔC, LRP6ΔE or LDLR could down-regulate YAP and CYR61 expressions, whereas only LRP6-ΔN did not influence YAP expression (Fig. 4G). In a nutshell, it was implicated that the LRP6 extracellular region, especially the LDLR region, potentially mediates the regulation of YAP.

Previous studies found that LRP6 could inhibit Wnt/PCP pathway^24–26^. It was thought that LDLR, the extracellular segment of LRP6, directly binds to the non-canonical Wnt pathway receptor FZD, as a mechanism to inhibit its mediated PCP signaling^26^. Herein, we were prompted to speculate that LRP6 might bind to FZD7 and disrupt its mediated PCP-YAP signaling. This hypothesis was supported by the phenomenon that the elevated expressions of phos-c-Jun and YAP induced by LRP6 silencing were rescued via FZD7 knockdown (Fig. 4E, H). In addition, we validated the interaction of LRP6 with FZD7, which was inhibited by cholesterol (Fig. 4I).

Collectively, these findings imply that LRP6 inhibits FZD7-mediated PCP-YAP signaling, potentially via its direct binding to FZD7. However, such interaction is impeded by cholesterol in colon cancer cells.

### Nystatin synergizes with targeting SOAT1 in suppressing the viability of colon cancer cells

Nystatin is a polyene antifungal drug for managing cutaneous or mucosal candidiasis^27^. Nystatin could directly bind to cholesterol and inhibit its functions, particularly disrupting cell membrane structure and lipid raft stability. Previous reports had demonstrated the potential role of nystatin in enhancing the anti-tumor effect of cetuximab and endostatin via cholesterol sequestration^28–30^.

**Fig. 5:**
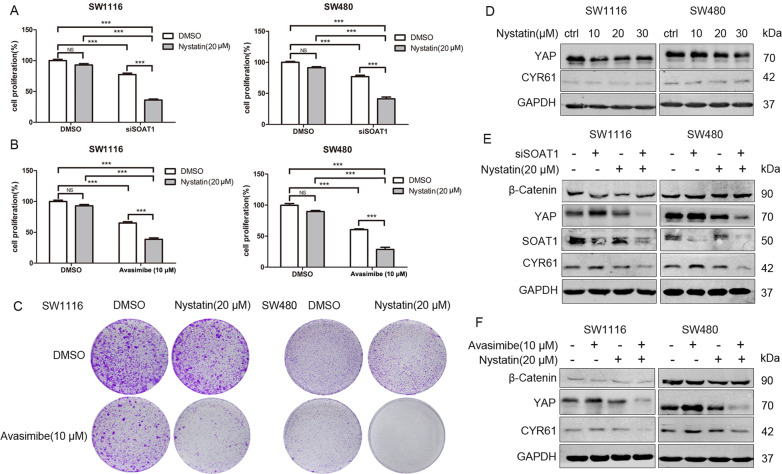
Targeting SOAT1 synergizes with nystatin in suppressing the viability of colon cancer cells in vitro. **A** SW1116 and SW480 were treated with control siRNA or siSOAT1 in the presence or absence of nystatin (20 μM). Cell proliferation activity was detected by CCK-8 after 72 h. **B** SW1116 and SW480 were treated with DMSO or avasimibe (10 μM) in the presence or absence of nystatin (20 μM). Cell proliferation activity was detected by CCK-8 after 72 h. **C** SW1116 and SW480 were incubated with DMSO or avasimibe (10 μM) in the presence or absence of nystatin (20 μM) for 5 d, respectively. The cell proliferation ability was detected by the colony formation assay. **D** SW1116 and SW480 were treated with nystatin (10–30 μM) for 16 h. **E** SW1116 and SW480 were transfected with negative control siRNA or SOAT1 siRNA for 48 h, respectively. The cells were then incubated with DMSO or nystatin (20 μM) for 16 h. **F** SW1116 and SW480 were incubated with DMSO or avasimbie (10 μM) in the presence or absence of nystatin (20 μM) for 16 h, respectively. The expression of proteins was detected by Western blot.

Cholesterol-regulated signaling potentially mediates the resistance of colon cancer cells to SOAT1 inhibition. Thus, nystatin-mediated cholesterol sequestration may sensitize cells to SOAT1 inhibition. We found that high nystatin concentration significantly induced cell death in colon cancer cells, which could be partially rescued by cholesterol as a supplement (Fig. S6). As intravenous administration of nystatin tends to induce acute liver injury and renal toxicity, we administered a low dose of nystatin in our experimental conditions. Although the CCK-8 and colony formation assays demonstrated that a low dose of nystatin did not significantly impact the viability of SW1116 and SW480 cells, it could sensitize cells to SOAT1 knockdown or avasimibe, obviously (Fig. 5A–C).

Mechanistically, we found that nystatin had no obvious effect on YAP expression (Fig. 5D); however, it significantly lowered YAP expression under avasimibe or SOAT1 siRNA in colon cancer cells (Fig. 5E, F). Unexpectedly, no significant decrease in β-Catenin expression after the combined treatment was reported. Also, β-Catenin expression was not up-regulated via SOAT1 silencing or avasimibe; this implied that the canonical Wnt/β-Catenin signaling might be suffered to a more complex regulation following SOAT1 inhibition and nystatin treatment (Fig. 5E, F). Similar to the nystatin effect, direct inhibition of YAP function by verteporfin promoted the inhibitory effect of avasimibe on the survival of SW480 and SW1116 cells (Fig. S7). Moreover, restoration of YAP expression rescued the inhibition of cell survival induced by the combination of avasimibe and nystatin (Fig. S8). These findings provide evidence that cholesterol-regulated YAP signaling mediated the resistance of colon cancer cells to SOAT1 inhibition.

**Fig. 6:**
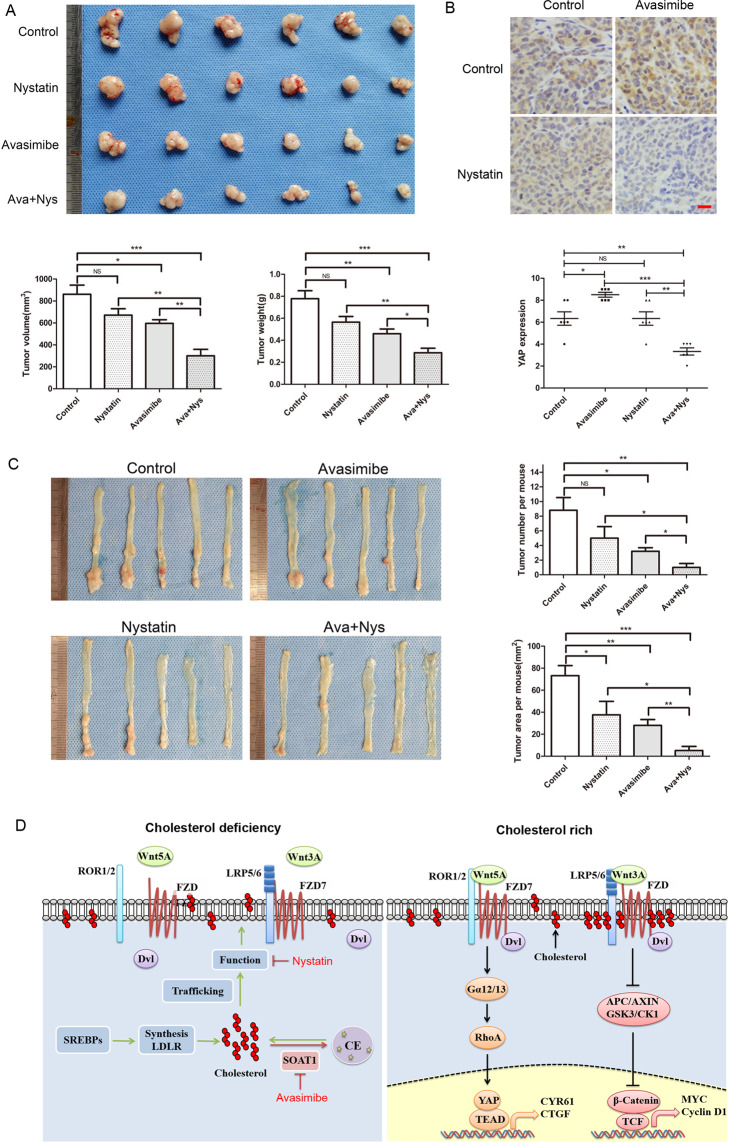
Targeting SOAT1 synergizes with nystatin in suppressing the viability of colon cancer cells in vivo. **A** SW480 cells were implanted subcutaneously in nude mice, and randomly divided into 4 groups (*N* = 6). Each group was intraperitoneal injection with control solvent (normal saline, PEG300, and DMSO), avasimibe (15 mg/kg.d), nystatin (4 mg/kg.d) and combined drugs. After 4 weeks, the xenografts were measured and weighed. The bar graphs indicate tumor volume and weight of each group. **B** Representative pictures of YAP staining in xenograft tumor tissues. The graph indicates the IHC scores of YAP expression. Scale bar: 20 μm. **C** Mouse models of AOM/DSS-induced colorectal cancer were described in ‘Materials and methods’ section. The mice were randomly divided into 4 groups (*N* = 5), each group was intraperitoneally injected with control solvent, avasimibe (15 mg/kg.d), nystatin (4 mg/kg.d) and combined drugs during the period of drinking normal water. The graphs showed the average number and tumor area of each group. Statistical methods: one-way ANOVA followed by Tukey post hoc test, *p < 0.05, **p < 0.01, ***p < 0.001, NS indicates no significant difference. **D** The model of regulation of YAP by cholesterol in colon cancer. LRP6 is released from the cholesterol-rich membranes (lipid rafts) and directly binds to FZD7, resulting in the inactivation of PCP-YAP pathway and classical Wnt pathway when lack of cholesterol. After membrane cholesterol levels increased, LRP6 is transfered to cholesterol-rich area, alleviating the inhibitory effect of LRP6 on PCP-YAP pathway, and activating classical Wnt pathway simultaneously.

To further validate the synergistic effect of avasimibe and nystatin, we performed in vivo experiment in the xenograft model of SW480 cells. Results demonstrated that nystatin synergized with avasimibe in suppressing the tumor growth (Fig. 6A). IHC analysis revealed that avasimibe slightly elevated YAP expression in the xenografts, which was significantly lowered following combined treatment (Fig. 6B). When we injected avasimibe and nystatin in the mouse models of AOM/DSS-induced inflammation-associated colorectal cancer, nystatin synergized with avasimibe in suppressing the formation and growth of the tumor (Fig. 6C). Notably, the body weights of the xenograft model and the AOM/DSS mouse model exhibited no statistical difference between groups (Fig. S9).

## Discussion

### The interplay between the Hippo pathway and cholesterol metabolism

There exist an interplay between the Hippo pathway and metabolism. Metabolic factors, including glucose and hormones, have been revealed to regulate the Hippo pathway. Conversely, the Hippo pathway also regulates metabolic processes, such as glycolysis and glutaminolysis^31^. Although the direct evidence regarding the regulation of cholesterol metabolism by YAP/TAZ is still lacking, LATS2 has been reported to inhibit SREBP activity and suppress hepatic cholesterol accumulation^32^.

MVA pathway is the first to be reported as a link between the Hippo pathway and cholesterol metabolism^17^. Geranylgeranyl diphosphate, an intermediate metabolite of the MVA pathway, is utilized for protein geranylgeranylation, an essential modification for RhoA membrane anchorage and activation. RhoA inhibits LATS activity, activating YAP, potentially by modulating the actin cytoskeleton or activating ROCK1/2; however, the specific mechanism is yet to be elucidated^33^. Bile acids, the derivatives of cholesterol, also promotes YAP activation by down-regulating the expressions of MST and LATS^34^.

In recent years, increasing number of studies have implicated cholesterol as an important signaling molecule, regulating several pathways. For instance, cholesterol could enhance the signal transduction of various membrane receptors, such as integrin and c-Met, by maintaining lipid rafts stability^35,36^. Cholesterol modification of Hedgehog and Smoothened is crucial in the activation of Hedgehog signaling^19,20,37^. Besides, cholesterol exhibit a high affinity to Dishevelled (Dvl), promoting its membrane localization and activating the canonical Wnt pathway^18^. Another study revealed that cholesterol was an endogenous ligand of ERRα, a critical regulator of multiple cancers^38^. Recently, increased hepatocyte cholesterol was found to block the proteasomal degradation of TAZ, the core protein in the Hippo pathway via the adenylyl cyclasee-RhoA pathway, and promotes fibrotic non-alcoholic steatohepatitis^39^.

In the present study, we revealed that intracellular cholesterol could promote YAP expression via the FZD7/PCP/RhoA pathway in colon cancer cells. Notably, this validated the regulatory effect of cholesterol on the Hippo/YAP pathway. Contrary to previous reports, LATS activity is not decreased post cholesterol treatment, demonstrating that cholesterol-RhoA signaling potentially regulates YAP via alternative LATS independent mechanisms in colon cancer cells^40,41^.

### Cholesterol coordinated Wnt signaling

With the discovery of 19 Wnt proteins and over 15 receptors and co-receptors, the signal transduction of Wnt pathways (canonical Wnt pathway, PCP pathway, and Wnt/Ca^2+^ pathway) is extremely complex. PCP signaling and canonical Wnt signaling are known for their antagonistic characteristics, whereby inhibiting one generally up-regulates the other^42^. For example, Wnt5A could competitively inhibit Wnt3A binding to FZD2, consequently suppressing the canonical Wnt signaling upon activation of the PCP pathway^43^. Moreover, the canonical Wnt signaling promotes the assembly of active LRP5/6-FZD receptor complexes that recruit Dvl and actively impede the contribution of Dvl in the PCP signaling^25^. LRP5/6 can also directly bind to FZD receptors in a Wnt-independent manner, subsequently inhibiting the FZD-mediated PCP pathway^26^.

Canonical Wnt and PCP pathways both play critical roles in tumor formation and progression^44^. The mechanisms for alleviating the antagonistic effects of these two pathways in cells are suggested to be vital for tumors, although they remain elusive. In this study, we found that cholesterol could activate the canonical Wnt and PCP pathways, simultaneously. Mechanistically, cholesterol alleviated the inhibitory effect of LRP6 on the FZD7-PCP pathway, thereby promoting PCP-RhoA-YAP signaling. Similarly, the dissociation of LRP6-FZD7 induced by cholesterol may also promote the assembly of canonical Wnt receptor complexes, thus inducing canonical Wnt signaling.

Of note, canonical Wnt signaling triggers caveolin-mediated endocytosis; here, the LRP5/6-FZD receptor complexes are essentially localized in lipid rafts^45–47^. A wealth of reports demonstrated that blocking caveolin-mediated endocytosis of LRP5/6 inhibits canonical Wnt signaling^46,48^. Conversely, the localization of ROR1/2-FZD receptor complexes in non-lipid raft domains and clathrin-mediated endocytosis may potentiate PCP signaling^43,49,50^. Therefore, it is conceivable that cellular cholesterol, especially membrane cholesterol may promote LRP5/6 transfer from non-lipid raft domains to lipid rafts, perhaps by stabilizing lipid rafts or via direct binding; subsequently, the canonical Wnt pathway is activated. Meanwhile, this transfer inhibits the association of LRP5/6 with FZD7 in non-lipid raft domains; as a result, FZD7 is release to mediate the PCP pathway (Fig. 6D). In summary, the above findings demonstrated a novel function of cholesterol in coordinating Wnt signaling, which is potentially vital for tumor progression.

### Targeting SOAT1 in cancer

Sterol O-acyltransferase (SOAT1/2) catalyzes the formation of cholesteryl esters from cholesterol and long-chain fatty acids in cells^7^. Notably, several studies have reported high SOAT1 expression in prostate cancer, pancreatic cancer, malignant glioma, and some other tumors, accompanied by high cholesteryl esters content^3,9,10,12^. Although the mechanisms driving the high SOAT1 expression in tumors remain elusive, targeting SOAT1 has proved to be a promising therapeutic strategy for managing cancers.

In malignant glioma and prostate cancer, avasimibe-mediated SOAT1 inhibition could elevate intracellular cholesterol content, consequently impeding SREBP1 activity and SREBP1-modulated fatty acids synthesis and uptake^10,12^. Additionally, avasimibe could promote the apoptosis of pancreatic cancer cells via cholesterol-induced ER stress^9^. Hepatocellular carcinoma characterized by high levels of SOAT1 expression was found to be associated with poor prognosis; notably, avasimibe could exert a therapeutic effect in such tumors^3^. Also, targeting SOAT1 via avasimibe could enhance the killing effect of CD8 + T cells on melanoma by elevating membrane cholesterol content; thus, promoting T-cell receptor (TCR) aggregation and immune synapse formation^13^.

As previously reported, increased intracellular cholesterol content may induce the inhibitory effect of targeting SOAT1 by blocking lipid metabolism. However, some oncogenic signaling may be promoted by increased cholesterol content, especially in the membrane, that potentially desensitizes cancer cells to avasimibe. In the present study, we found that targeting SOAT1 could activate the PCP-YAP axis in a cholesterol-dependent manner, whereas nystatin sensitized cancer cells to avasimibe via cholesterol sequestration. Interestingly, nystatin could solely inhibit YAP expression under SOAT1 inhibition. It is supposed that the compensatory pathways, including the MVA pathway, potentially are enhanced to maintain YAP expression post nystatin treatment; notably, this could be reversed by targeting SOAT1. Meanwhile, targeting SOAT1 may not only promote nystatin binding to cells but also block the update of cholesterol in the membrane following nystatin binding. Elevated membrane cholesterol content might also promote other oncogenic signaling. Thus, nystatin-driven cholesterol inhibition should be superior to the inhibition of the PCP-YAP pathway.

In conclusion, the present study uncovered a novel function of cholesterol in PCP-YAP pathway regulation. Notably, cholesterol alleviates the inhibitory effect of LRP6 on the PCP pathway by impeding the association of LRP6 with FZD7. Also, targeting SOAT1 promotes YAP expression by elevating cellular cholesterol content in colon cancer cells, whereas nystatin-mediated cholesterol sequestration inhibits YAP expression in the absence of SOAT1. In vitro and in vivo results further, demonstrate that nystatin synergizes with avasimibe-associated SOAT1 targeting in suppressing the viability of colon cancer cells. Collectively, these findings offer a novel strategy for cholesterol metabolic-targeted treatment of colon cancers.

## Materials and methods

### Cell culture and reagents

SW1116, SW480 were originally obtained from ATCC and reserved in our laboratory. Filipin III (Santa Cruz), Bodipy 493/503 (Thermo Fisher), Cholesterol, β-Cyclodextrin, and AOM (Sigma), Avasimibe and LGK974 (Selleck), Nystatin (MCE), DSS (MP Biomedicals). Antibody against YAP, pYAPSer127, β-Catenin, c-Myc, FZD8, RhoA, c-Jun, GST were all from Abcam. Antibody against LRP6, pLRP6Ser1490, GSK-3β, pGSK-3βSer9, pLATS1Ser909, LATS1 were from CST. Antibody against pc-JunSer63, FZD1, CYR61 were from Sangon Biotech. Antibody against LDLR, HMGCR, SOAT2, FZD2, FZD5, FZD7, Myc were from Proteintech. Anti-SOAT1 was from Abclonal. Anti-GAPDH was from ZSGB-Bio.

### Filipin III and Bodipy 493/503 staining

Cells were fixed with 4% paraformaldehyde for 15 min following 1.5 mg/mL Glycine for 30 min. Rinse cells three times with PBS. Cells were incubated with 100 μg/mL Filipin III for 2 h, or 1 μg/mL Bodipy 493/503 for 30 min at room temperature. Immunofluorescence was detected using fluorescence microscope (Eclipse 80i, Nikon) at ×200 magnifications. The quantitative result of cellular cholesterol content was analyzed by Cholesterol Assay Kit (Abcam) according to the protocol.

### Oil Red O staining

Prepare fresh-frozen tissue sections. Incubate slide in 100% isopropanol for 5 min. Incubate slide in Oil Red O solution for 10 min. Differentiate section in 85% isopropanol for 3 min. Rinse slide twice in water. Incubate in hematoxylin for 1–2 min, rinse slide three times in water. Coverslip with glycerogelatin. The fresh-frozen human colon cancer tissues were obtained from Biological Specimen Bank of West China Hospital. All the patients signed informed consent forms. This study was approved by the Ethics Committee of West China Hospital. The percentage of lipid droplets positive cells was scored as 0 (<1%), 1 (1–25%), 2 (26–50%), 3 (51–75%), or 4 (>75%).

### Transfection

SiRNAs targeting SOAT1, RhoA, β-Catenin, LRP5/6, FZD1/2/5/7/8, and negative control were synthesized by GenePharma. The sequences of siRNAs used in this study are provided in the supplementary file. LRP6 and deletion mutants vectors were gifts from Prof. Weidong Zhu (Tongji University School of Medicine, China). FZD7 plasmid was obtained from Public Protein/Plasmid Library. Lipofectamine 2000 (Invitrogen) was used for transfection according to the manufacturer’s instructions. The lentiviral vector expressing shLRP6#UTR was constructed by Vigenebio, and was transfected in cells according to the protocol.

### RhoA pull-down assay

Glutathione S-transferase (GST)-fusion peptides (Rhotekin Rho-binding domain) were expressed in E.coli BL21 and purified by glutathione beads (Sigma). Cells of each group were lysed in cell lysis buffer. Transfer the supernatant to a new tube. Add 40 µL of resuspended beads with the bound proteins to the tube. Incubate the tube at 4 °C for 1 h with gentle agitation. Wash the bead 3 times with cell lysis buffer. Pellet the beads and carefully remove all the supernatant. Resuspend the beads in 40 µL of 2X SDS-PAGE buffer. Boil each sample for 5 min. Centrifuge each sample, and use the supernatant to perform western blot analysis.

### RNA extraction, cDNA synthesis, and Quantitative Real-Time PCR

PCR primers used in this study are provided in Fig. S4. Gene expression levels for genes of interest were normalized to GAPDH and calculated as ΔC_T_ values (ΔC_T_ = C_T_ gene of interest–C_T_ GAPDH). Log2 fold changes in expression between treatment group and control group were calculated using the formula: log2 fold change = –ΔΔC_T_ = –[ΔC_T_ treatment group – ΔC_T_ control group]. RNA extraction and cDNA synthesis were performed according to the protocol.

### Immunoprecipitation assays

Cells were lysed with Immunoprecipitation (IP) lysis buffer (Beyotime). The lysate was then incubated with antibody against LRP6 overnight at 4°C. Precipitate the antibody-protein complex by protein A/G magnetic beads (Selleck). The immunoprecipitates were washed five times, and then subjected to Western Blotting analysis.

### In vivo xenograft experiments

Female BALB/c nude mice (5–6 weeks old, from HFK Bioscience) were subcutaneously injected with SW480 cells (1.0×10^7^). Ten days after implantation, mice with tumors were randomly assigned to four groups (*n* = 6), and intraperitoneal injected with control solvent (normal saline, PEG300, and DMSO), avasimibe (15 mg/kg.d), nystatin (4 mg/kg.d), and combined drugs for 28 days. Tumor volumes were calculated using the following formula: *V* = (*L*×*W*^2^)/2, where *L* and *W* represent length and width. Details of cell proliferation assays and colony formation assays in vitro were described in our previous report^15^.

### Mouse models of AOM/DSS-induced colorectal cancer

Male C57BL/6 mice (5–6 weeks old, from HFK Bioscience) were intraperitoneally injected with azoxymethane (AOM, 10 mg/kg) at day 1. Fill the water bottles with 1.25% (wt/vol) dextran sulfate sodium (DSS)-containing water for 7 days. Empty the remaining DSS solution from the bottles at day 8 and refill with normal water. Two weeks later, fill with DSS solution for another 7 days. Three cycles of DSS treatment were needed during the progress. At day 30, mice were randomly assigned to four groups (*n* = 5), and intraperitoneal injected with control solvent (normal saline, PEG300, and DMSO), avasimibe (15 mg/kg.d), nystatin (4 mg/kg.d), and combined drugs during the period of drinking normal water. After 3 months, the mice were sacrificed and dissected, and the tumors were counted and measured. All experiments regarding nude mice and C57BL/6 mice were performed in accordance with the institute guidelines and were approved by the animal ethics committee of the China Institute of Science.

### Colon cancer tissue microarray

The human colon cancer tissue microarrays were prepared by Shanghai Outdo Biotech, China. All the patients signed informed consent forms. This study was approved by the Ethics Committee of Taizhou Hospital of Zhejiang Province. IHC was performed on human colon cancer samples and xenograft tumor tissues according to the protocol. IHC score was based on the multiplied result of percentage positivity and staining intensity. The percentage of positive cells was scored as 0–4 (<1%, 1–25%, 26–50%, 51–75%, >75%). Staining intensity was scored as 0–3 (no staining, weak staining, moderate staining, strong staining).

### Statistical analysis

Student’s *t* test (two-tailed) and one-way ANOVA followed by Tukey post hoc test were used for comparison between groups by GraphPad Prism 5. Statistical significance was set at *p < 0.05, **p < 0.01, and ***p < 0.001. No statistical methods were used to predetermine sample size. All experiments were performed using at least three biological replicates.

## Supplementary information

Supplementary figure S1

Supplementary figure S2

Supplementary figure S3

Supplementary figure S4

Supplementary figure S5

Supplementary figure S6

Supplementary figure S7

Supplementary figure S8

Supplementary figure S9

Supplementary file of sequences

Supplementary figure legends

## References

[1] Ward PS, Thompson CB (2012). Metabolic reprogramming: a cancer hallmark even warburg did not anticipate. Cancer Cell.

[2] Maxfield FR, Tabas I (2005). Role of cholesterol and lipid organization in disease. Nature.

[3] Jiang Y (2019). Proteomics identifies new therapeutic targets of early-stage hepatocellular carcinoma. Nature.

[4] Wang B (2018). Phospholipid remodeling and cholesterol availability regulate intestinal stemness and tumorigenesis. Cell Stem Cell.

[5] Gallagher EJ, Zelenko Z, Neel BA, Antoniou IM, Leroith D (2017). Elevated tumor LDLR expression accelerates LDL cholesterol-mediated breast cancer growth in mouse models of hyperlipidemia. Oncogene.

[6] Moon S, Huang C, Houlihan SL, Prives C (2019). p53 represses the mevalonate pathway to mediate tumor suppression. Cell.

[7] Chang T, Chang CCY, Ohgami N, Yamauchi Y (2006). Cholesterol sensing, trafficking, and esterification. Annu. Rev. Cell Dev. Biol..

[8] Catherine CYC (2000). Immunological quantitation and localization of ACAT-1 and ACAT-2 in human liver and small intestine. J. Biol. Chem..

[9] Li J (2016). Abrogating cholesterol esterification suppresses growth and metastasis of pancreatic cancer. Oncogene.

[10] Yue S (2014). Cholesteryl ester accumulation induced by PTEN Loss and PI3K/AKT activation underlies human prostate cancer aggressiveness. Cell Metab..

[11] Xu H, Zhou S, Tang Q, Xia H, Bi F (2020). Cholesterol metabolism: new functions and therapeutic approaches in cancer. Biochim. Biophys. Acta Rev. Cancer.

[12] Geng F (2016). Inhibition of SOAT1 suppresses glioblastoma growth via blocking SREBP-1-mediated lipogenesis. Clin. Cancer Res..

[13] Yang W (2016). Potentiating the antitumour response of CD8(+) T cells by modulating cholesterol metabolism. Nature.

[14] Zhao B, Tumaneng K, Guan K (2011). The Hippo pathway in organ size control, tissue regeneration and stem cell self-renewal. Nat. Cell Biol..

[15] Xu H (2019). MEK nuclear localization promotes YAP stability via sequestering *β*-TrCP in KRAS mutant cancer cells. Cell Death Differ..

[16] Ye J, DeBose-Boyd RA (2011). Regulation of cholesterol and fatty acid synthesis. Cold Spring Harb. Perspect. Biol.

[17] Sorrentino G (2014). Metabolic control of YAP and TAZ by the mevalonate pathway. Nat. Cell Biol..

[18] Sheng, R. et al. Cholesterol selectively activates canonical Wnt signalling over non-canonical Wnt signalling. *Nat. Commun.* **5**, (2014). 10.1038/ncomms5393 PMC4100210 25024088

[19] Huang P (2017). Cellular cholesterol directly activates smoothened in hedgehog signaling. Cell.

[20] Xiao X (2017). Cholesterol modification of smoothened is required for Hedgehog signaling. Mol. Cell.

[21] Dai X (2015). YAP activates the Hippo pathway in a negative feedback loop. Cell Res..

[22] Konsavage WM, Kyler SL, Rennoll SA, Jin G, Yochum GS (2012). Wnt/*β*-catenin signaling regulates Yes-associated protein (YAP) gene expression in colorectal carcinoma cells. J. Biol. Chem..

[23] Park HW (2015). Alternative Wnt signaling activates YAP/TAZ. Cell.

[24] Bryja V (2009). The extracellular domain of Lrp5/6 inhibits noncanonical Wnt signaling in vivo. Mol. Biol. Cell.

[25] Tahinci E (2007). Lrp6 is required for convergent extension during Xenopus gastrulation. Development.

[26] Ren D (2015). LRP5/6 directly bind to Frizzled and prevent Frizzled-regulated tumour metastasis. Nat. Commun..

[27] Gøtzsche PC, Johansen HK (2014). Nystatin prophylaxis and treatment in severely immunodepressed patients. Cochrane Database Syst. Rev..

[28] Baek S, Kim SM, Lee SA, Rhim BY, Eo SK (2013). The cholesterol-binding antibiotic nystatin induces expression of macrophage inflammatory protein-1 in macrophages. Biomol. Therapeutics.

[29] Chen Y (2015). Enhancement of tumor uptake and therapeutic efficacy of EGFR-targeted antibody cetuximab and antibody-drug conjugates by cholesterol sequestration. Int. J. Cancer.

[30] Chen Y (2011). Cholesterol sequestration by nystatin enhances the uptake and activity of endostatin in endothelium via regulating distinct endocytic pathways. Blood.

[31] Koo JH, Guan K (2018). Interplay between YAP/TAZ and Metabolism. Cell Metab..

[32] Aylon Y (2016). The LATS2 tumor suppressor inhibits SREBP and suppresses hepatic cholesterol accumulation. Genes Dev..

[33] Mi W (2015). Geranylgeranylation signals to the Hippo pathway for breast cancer cell proliferation and migration. Oncogene.

[34] Anakk S (2013). Bile acids activate YAP to promote liver carcinogenesis. Cell Rep..

[35] Zeng J (2018). Aggregation of lipid rafts activates c-met and c-Src in non-small cell lung cancer cells. BMC Cancer.

[36] Jin H (2019). Targeting lipid metabolism to overcome EMT-associated drug resistance via integrin β3/FAK pathway and tumor-associated macrophage repolarization using legumain-activatable delivery. Theranostics.

[37] Gallet A, Rodriguez R, Ruel L, Therond PP (2003). Cholesterol modification of hedgehog is required for trafficking and movement, revealing an asymmetric cellular response to hedgehog. Dev. Cell.

[38] Wei W (2016). Ligand activation of ERRαby cholesterol mediates statin and bisphosphonate effects. Cell Metab..

[39] Wang X (2020). Cholesterol Sstabilizes TAZ in hepatocytes to promote experimental non-alcoholic steatohepatitis. Cell Metab..

[40] Zhang Y (2014). CD44 acts through RhoA to regulate YAP signaling. Cell. Signal..

[41] Huang Z (2016). RhoA deficiency disrupts podocyte cytoskeleton and induces podocyte apoptosis by inhibiting YAP/dendrin signal. BMC Nephrol..

[42] Niehrs C (2012). The complex world of WNT receptor signalling. Nat. Rev. Mol. cell Biol..

[43] Sato A, Yamamoto H, Sakane H, Koyama H, Kikuchi A (2010). Wnt5a regulates distinct signalling pathways by binding to Frizzled2. EMBO J..

[44] Anastas JN, Moon RT (2013). WNT signalling pathways as therapeutic targets in cancer. Nat. Rev. Cancer.

[45] Hideki Y, Hideyuki K, Akira K (2006). Caveolin is necessary for Wnt-3a-dependent internalization of LRP6 and accumulation of beta-catenin. Dev. Cell.

[46] Yamamoto H, Sakane H, Yamamoto H, Michiue T, Kikuchi A (2008). Wnt3a and Dkk1 regulate distinct internalization pathways of LRP6 to tune the activation of beta-catenin signaling. Dev. Cell.

[47] Bilic J (2007). Wnt induces LRP6 signalosomes and promotes dishevelled-dependent LRP6 phosphorylation. Science.

[48] Jiang Y, He X, Howe PH (2012). Disabled-2 (Dab2) inhibits Wnt/β-catenin signalling by binding LRP6 and promoting its internalization through clathrin. EMBO J..

[49] Ohkawara B, Glinka A, Niehrs C (2011). Rspo3 binds syndecan 4 and induces Wnt/PCP signaling via clathrin-mediated endocytosis to promote morphogenesis. Dev. cell.

[50] Kikuchi A, Yamamoto H, Sato A, Matsumoto S (2012). Wnt5a: its signalling, functions and implication in diseases. Acta Physiologica (Oxf., Engl.).

