# Supplementary figures and images for: Cholesterol activates the Wnt/PCP-YAP signaling in SOAT1-targeted treatment of colon cancer
Source: Cell Death Discov. 2021 Feb 26;7:38. doi: 10.1038/s41420-021-00421-3 (PMC7910478; doi:10.1038/s41420-021-00421-3)

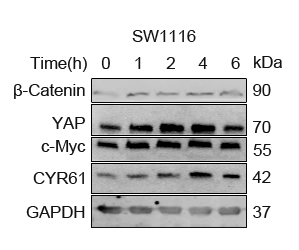

Supplement: Supplementary file 1 — Supplementary figure S1 [file 41420_2021_421_MOESM1_ESM.tif]

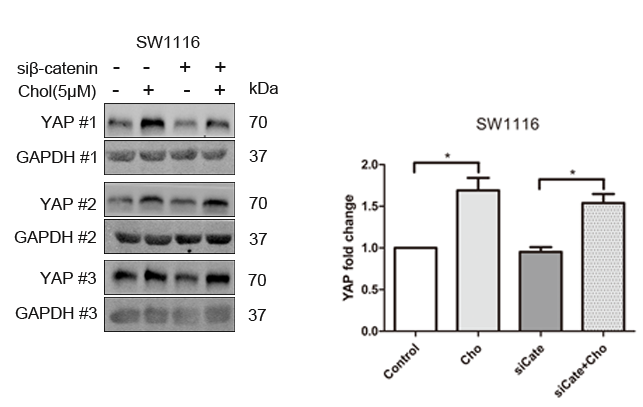

Supplement: Supplementary file 2 — Supplementary figure S2 [file 41420_2021_421_MOESM2_ESM.tif]

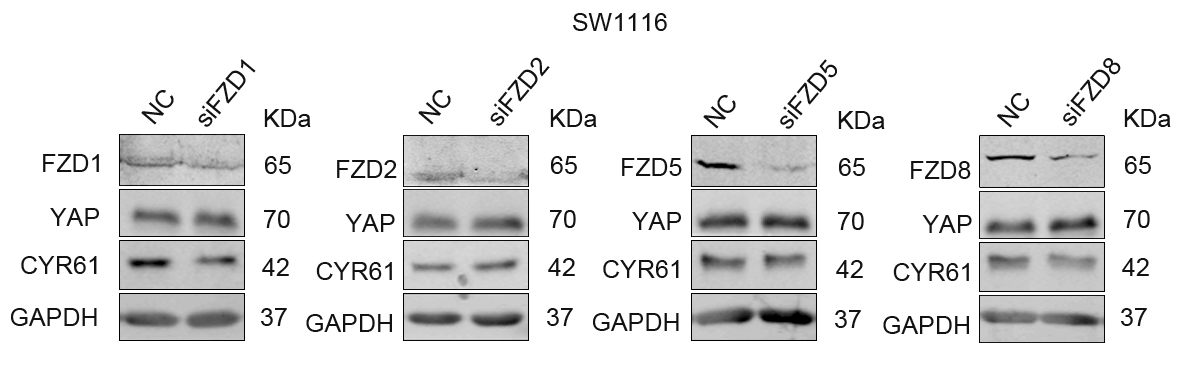

Supplement: Supplementary file 3 — Supplementary figure S3 [file 41420_2021_421_MOESM3_ESM.tif]

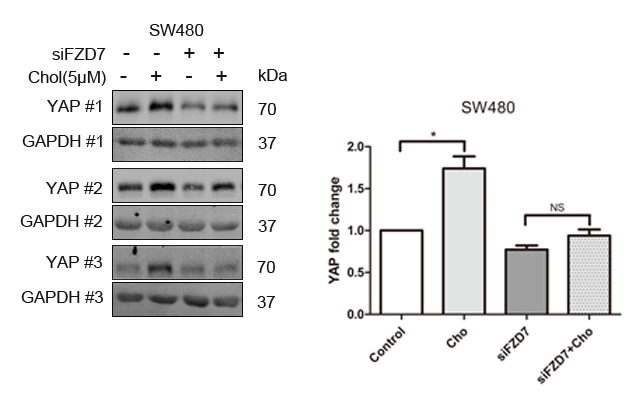

Supplement: Supplementary file 4 — Supplementary figure S4 [file 41420_2021_421_MOESM4_ESM.tif]

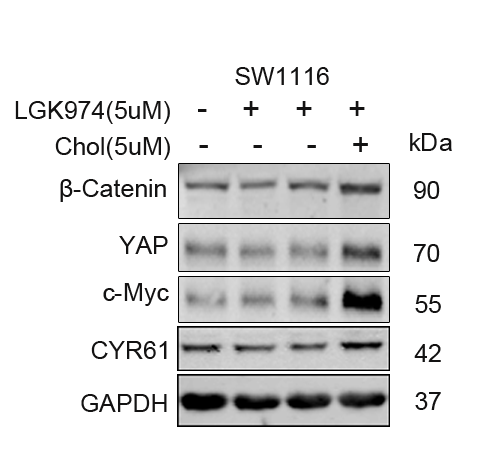

Supplement: Supplementary file 5 — Supplementary figure S5 [file 41420_2021_421_MOESM5_ESM.tif]

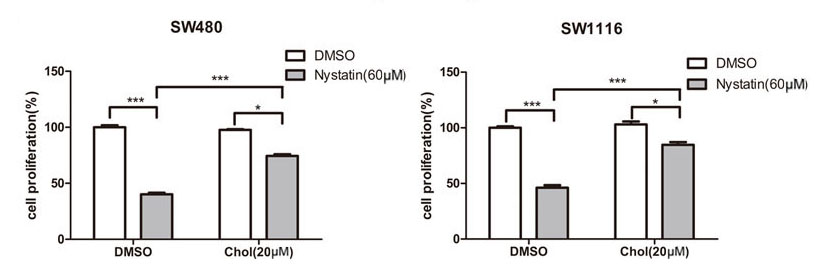

Supplement: Supplementary file 6 — Supplementary figure S6 [file 41420_2021_421_MOESM6_ESM.tif]

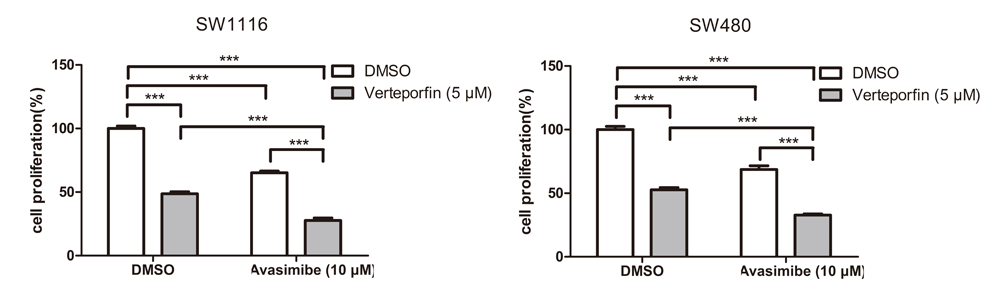

Supplement: Supplementary file 7 — Supplementary figure S7 [file 41420_2021_421_MOESM7_ESM.tif]

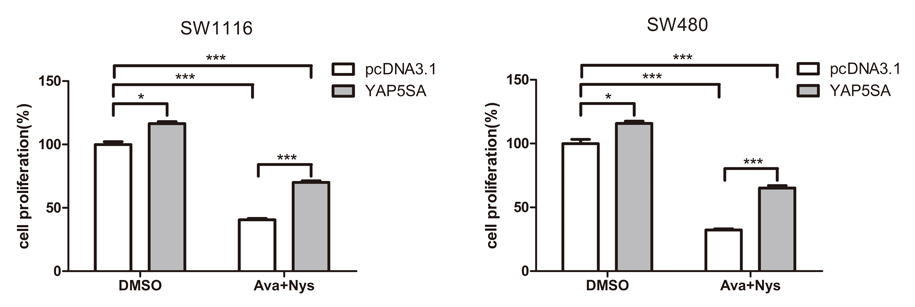

Supplement: Supplementary file 8 — Supplementary figure S8 [file 41420_2021_421_MOESM8_ESM.tif]

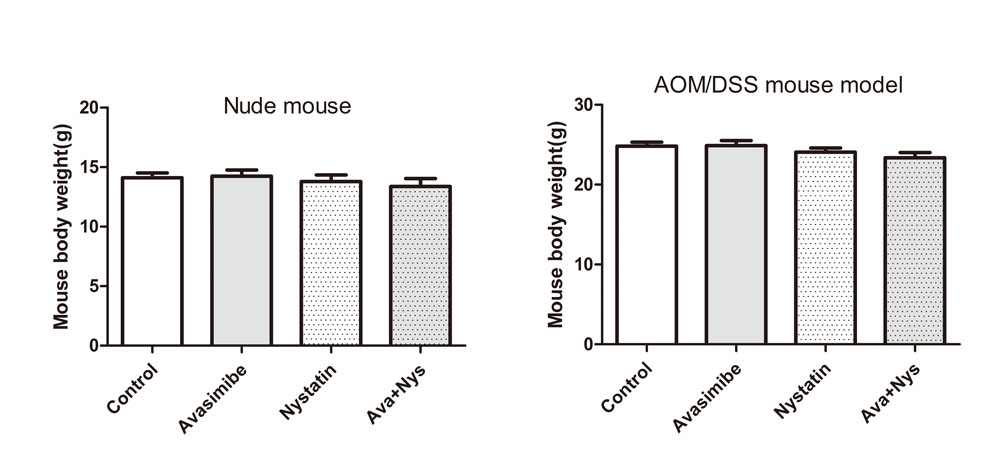

Supplement: Supplementary file 9 — Supplementary figure S9 [file 41420_2021_421_MOESM9_ESM.tif]

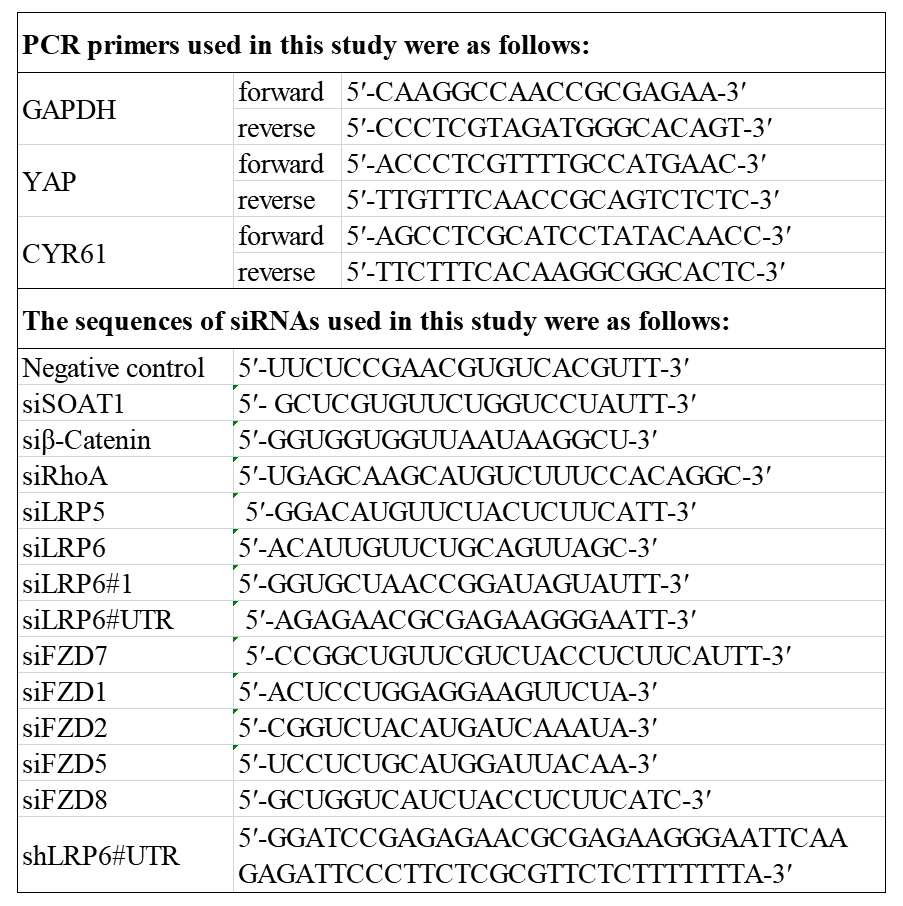

Supplement: Supplementary file 10 — Supplementary file of sequences [file 41420_2021_421_MOESM10_ESM.tif]
