# Supplementary material for: Cholesterol activates the Wnt/PCP-YAP signaling in SOAT1-targeted treatment of colon cancer
Source: Cell Death Discov. 2021 Feb 26;7:38. doi: 10.1038/s41420-021-00421-3 (PMC7910478; doi:10.1038/s41420-021-00421-3)
Supplement: Supplementary file 11 — Supplementary figure legends [file 41420_2021_421_MOESM11_ESM.docx]

Figure S1: SW1116 cells were starved in serum-free medium for 24 h, and then treated with cholesterol (5 μM) for different hours (0-6 h). The expressions of YAP, CYR61, β-Catenin and c-Myc were detected by Western blot.

Figure S2: SW1116 were transfected with negative siRNA or β-Catenin siRNA for 48 h, then starved for 24 h, and then incubated with DMSO or cholesterol (5 μM) for 2 h. The three independent western blot analysis was performed. The graph indicates YAP fold change relative to the control. Band intensities of YAP were quantified with the ODYSSEY Infrared Imaging System (LI-COR Biosciences), and normalized to the intensities of GAPDH. **p* < 0.05 using Student’s t test (two-tailed).

Figure S3: SW1116 cells were transfected with FZD1/2/5/8 siRNAs or negative control for 72 h, respectively.

Figure S4: SW480 were transfected with negative control siRNA or FZD7 siRNA for 48 h, and starved in serum-free medium for 24 h, then incubated with DMSO or cholesterol (5 μM) for 2 h, respectively. The three independent western blot analysis was performed. The graph indicates YAP fold change relative to the control. Band intensities of YAP were quantified with the ODYSSEY Infrared Imaging System (LI-COR Biosciences), and normalized to the intensities of GAPDH. **p* < 0.05, NS indicates no significant difference using Student’s t test (two-tailed).

Figure S5: SW1116 cells were starved in serum-free medium for 24 h, and then incubated with LGK974 (5μM) for 6 h, followed by DMSO or cholesterol (5 μM ) treatment for 2 h.

Figure S6: SW1116 and SW480 were treated with high concentration of nystatin (60 μM) for 16 h, then fresh medium containing cholesterol (20 μM) was replaced and incubated for 48 h. CCK-8 assays were performed to analyse the cell proliferation activity.

Figure S7: SW1116 and SW480 cells were treated with DMSO or avasimibe (10 μM) in the presence or absence of verteporfin (5 μM). Cell proliferation activity was detected by CCK-8 after 72 h.

Figure S8: SW1116 and SW480 cells were treated with DMSO or avasimibe (10 μM) combined with nystatin (20 μM) in the presence or absence of YAP5SA over-expression. The confirmation of YAP5SA over-expression was analyzed by WB in our previous study^15^. Cell proliferation activity was detected by CCK-8 after 72 h.

Figure S9: The bar graphs indicate the body weight of nude mouse and mouse model of colorectal cancer. The data presented as the mean±SD, showing no statistical significance between groups.

Table S1: The sequences of siRNAs and PCR primers used in this study.
